# Tonsillectomy May Not Be the Answer in All OSA Cases

**DOI:** 10.3390/jcm13216456

**Published:** 2024-10-28

**Authors:** Belén Bargagna, Carlos O’Connor-Reina, Laura Rodriguez-Alcala, Andrés Navarro, Gabriela Bosco, Nuria Pérez-Martín, Peter M. Baptista, Marina Carrasco-Llatas, Guillermo Plaza

**Affiliations:** 1 Otorhinolaryngology, Department, Hospital Quirónsalud Marbella, 29603 Málaga, Spain; 2 Sanatorio Güemes, Ciudad Autónoma de Buenos Aires, CP 3933 Buenos Aires, Argentina; 3 Otorhinolaryngology Department, Hospital Universitario de Fuenlabrada, Universidad Rey Juan Carlos, CP 28042 Madrid, Spain; 4 Otorhinolaryngology Department, Hospital Universitario Sanitas La Zarzuela, CP 28023 Madrid, Spain; 5 Otorhinolaryngology Department, Otorhinolaryngology Dep., Al Zahra Hospital, Dubai 124412, United Arab Emirates; 6 Otorhinolaryngology Department, Hospital Doctor Peset, CP 46017 Valencia, Spain

**Keywords:** tonsillectomy, sleep apnea, drug-induced sleep endoscopy

## Abstract

Tonsillectomy is considered the standard of care in patients with obstructive sleep apnea (OSA) and large tonsils; however, there are selected cases where this procedure should not be considered. We present two patients with tonsil grade 4 and severe OSA where tonsillectomy was not the solution for their problem and could be a superfluous procedure. In our experience, a preoperatory drug-induced sleep endoscopy (DISE) and proper patient phenotyping will prevent this type of surgical failure.

## 1. Introduction

Sleep-disordered breathing (SDB) is a widespread condition with substantial health consequences and continuously rising incidence [1]. Obstructive sleep apnea (OSA) is a prevalent form of SDB characterized by partial or complete obstruction of the upper airway. This obstruction results from the interaction of several key anatomical features and neuromuscular control of the upper airway (UA) [2].

**Figure 1:**
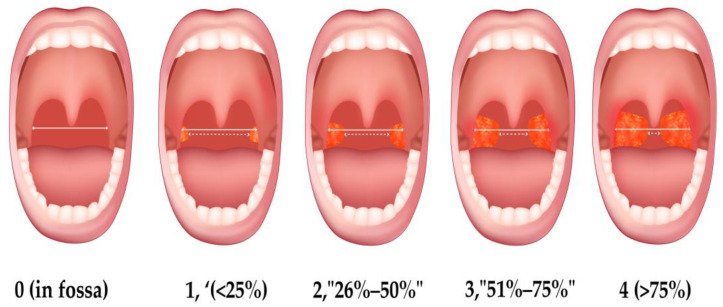
Friedman–Brodsky scale: grade 0: tonsils are entirely within the tonsillar fossa or have been removed (post-tonsillectomy); grade 1: tonsils occupy less than 25% of the oropharyngeal width; grade 2: tonsils occupy 25–50% of the oropharyngeal width; grade 3: tonsils occupy 50–75% of the oropharyngeal width; grade 4: tonsils occupy more than 75% of the oropharyngeal width, often referred to as “kissing” tonsils due to their proximity.

Tonsillar hypertrophy (TH) is one of the leading causes of snoring and OSA; it can be classified according to size (Friedman–Brodsky scale) [1,3] in four grades (Figure 1), with grades 3 and 4 being the main surgical indication for tonsillectomy (TA). This scale is significant in assessing patients with OSA because larger tonsils contribute to UA blockage. These anatomical findings contribute to sleep surgeons anticipating good results in their procedures and offering invitations for surgical treatment to patients with OSA [3]. As far as we know, no information has been published about the potential limitations of selecting adult patients with OSA and TH for upper airway surgery (UAS).

**Figure 2:**
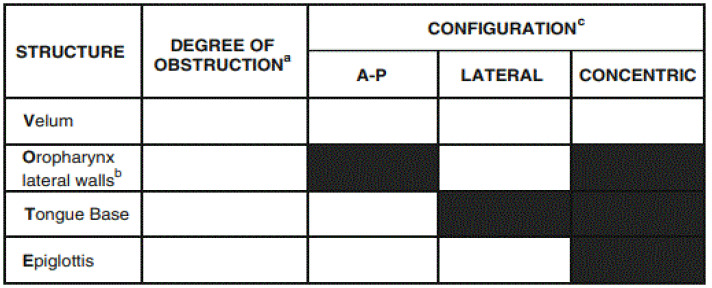
The surgical protocol used to code the DISE results according to the VOTE scale is as described in reference. ^a^ Degree of obstruction has one number for each structure: 0, No obstruction (no vibration); 1, Partial obstruction (vibration); 2, Complete obstruction (collapse); X, Not visualized. ^b^ Oropharynx obstruction can be distinguished as related solely to the tonsils or including the lateral walls, with or without a tonsillar component. ^c^ Configuration noted for structures with degree of obstruction greater than.

Drug-induced sleep endoscopy (DISE) [4] is considered in patients with persistent, post-adenotonsillectomy obstructive sleep apnea who refuse or fail positive pressure therapy. DISE is commonly used to explain anatomical findings in patients with OSA without a reasonable explanation of their disease. Vote classification is the most extended classification to evaluate the results from a DISE. The configuration of obstruction can be described as anteroposterior (typically anterior structures moving posteriorly against the posterior pharyngeal wall), lateral (laterally located structures moving towards the center of the airway), or concentric (a combination of the former two) [5], as seen in Figure 2.

Myofunctional therapy (MT) is based on a series of exercises performed with oropharyngeal muscles in order to establish proper tongue position and balanced strength vector forces in the stomatonagthic system [6]. Our group apply it to all patients with functional disorders following our published protocol [7].

Tonsillectomy is widely considered the gold standard treatment for children with OSA and large tonsils. Tonsillar hypertrophy is the leading cause of airway obstruction in pediatric OSA, and adenotonsillectomy is considered highly effective [8].

Adult tonsillectomy alone has a beneficial effect in OSA management, particularly in young overweight men with large tonsils, moderate OSA, and a low Friedman stage [9].

**Figure 3:**
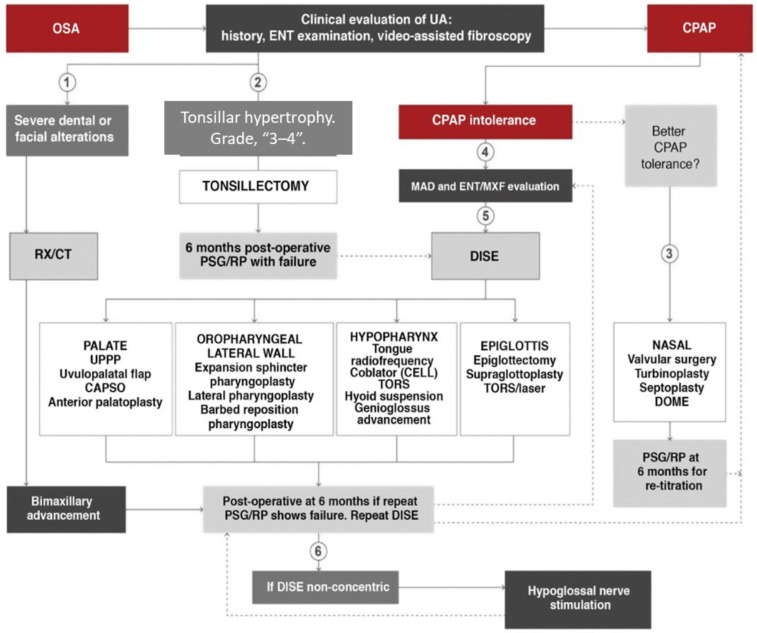
The indication algorithm for surgical treatment of obstructive sleep apnea (OSA) in adults follows precise and customized medicine. However, TA is recommended without previous DISE. (1) In case of severe dental or facial alterations, bimaxillary advancement may be indicated directly, although CPAP will be prescribed until surgery is conducted. (2) In case of tonsillar hypertrophy grade ≥3, tonsillectomy may be indicated directly, although CPAP will be prescribed until surgery. (3) In case of CPAP intolerance after ORL evaluation, nasal surgery is recommended as soon as possible to improve intolerance and re-titrate CPAP. (4) In case of CPAP intolerance after dental/maxillofacial evaluation, an adaptation of MAD or assessment for orthognathic surgery is recommended. (5) In case of CPAP intolerance after ENT evaluation, DISE is recommended to determine the most appropriate upper airway treatment. (6) After intolerance to CPAP and failure of other surgeries, a repeat DISE is recommended. If collapse is non-concentric, hypoglossal neurostimulation may be indicated. CAPSO: cautery-assisted palatal stiffening operation; CELL: coblator endoscopic lingual lightening; CPAP: continuous positive airway pressure; DISE: drug-induced sleep endoscopy; DOME: distraction osteogenesis maxillary expansion; ENT/MXF; ear, nose, and throat/maxillofacial; MAD: mandibular advancement device; PSG: polysomnography; RP: respiratory polygraph; RX/CT: radiological studies; TORS: transoral robotic surgery; UA: upper airway; UPPP: uvulopalatopharyngoplasty.

In the latest International Spanish Society of Pulmonology and Thoracic Surgery consensus on OSA, a therapeutic algorithm (Figure 3) was developed that allows direct surgical indication in adults with OSA (Apnea–Hypopnea Index [AHI] > 5) and grade 3–4 tonsils without requiring DISE before TA. This approach deviates from the traditional conception of surgery after failed continuous positive airway pressure (CPAP). It is centered on anatomical findings, classifying procedures according to the organ involved, and relying on diagnostic and exploratory findings to choose the most appropriate treatment [1], as suggested by Rotenberg et al. [10]. This study’s comprehensive analysis of the existing literature may offer new perspectives on surgical options, lifestyle interventions, or other non-invasive therapies that could be considered viable first-line treatments. By highlighting the benefits and limitations of different approaches, this paper challenges clinicians to rethink the traditional treatment paradigm and consider more personalized approaches to managing OSA.

However, the extent to which TH can be considered a cause of OSA and the effectiveness of TA as a stand-alone treatment for OSA have not been clarified. Existing publications do not provide comparable data, and the number of patients included is limited [2,10,11,12,13,14,15,16,17].

In some patients, SDB and OSA may persist or worsen after TA. This may be due to many factors influencing the cephalometric spacing of the UA: the size of the tonsils, the characteristics and function of the oropharyngeal muscles, the volume of the base of the tongue, epiglottis, and soft tissues and the position of the hyoid, among others [6,7,8,9,10,11,12].

Based on morphometry, there is a group of patients in which the palatine tonsils act as a kind of “stent” of the UA. In these cases, the tonsils support structures such as the base of the tongue or the lateral walls of the pharynx, and after their resection, a collapse of the UA may occur [18,19].

We aim to show three cases illustrating contrasting situations after TA in patients with OSA. In the first case, we observed that TA did not improve the OSA. In the second, we found that the palatine tonsils, rather than obstructing, may stabilize the UA by keeping it open and structurally supported. Lastly, in the third case, we found that TA and barbed pharyngoplasty did not improve OSA after 5 years and caused several prolonged minor oral side effects.

## 2. Case Reports

### 2.1. Case 1

The patient is a 24-year-old male who presented with snoring and apnea, reported by his partner, with daytime sleepiness and morning headache. Physical examination revealed a body mass index (BMI) of 25.1, Friedman 4, and grade 3 hypertrophy of the tonsils. A polygraph was requested, which showed a total recording time (TRT) of 6 h 12 min, an AHI of 80 events/h, predominantly obstructive (73.8), an Oxygen Desaturation Index (ODI) of 72.9, a baseline saturation of 98%, minimum saturation of 69%, and a cumulative percentage of the time spent below 90% O_2_ saturation (CT90) of 58%. According to the recommendation guidelines, TA was performed without prior sleep endoscopy, and the patient recovered favorably in the post-operative period without complications. Myofunctional evaluation showed normal IOPI scores: 67 kps in the maximum tongue strength and 30 kps in maximum lip strength.

After 12 months, the patient returned because of persistent snoring and apnea so a new polygraph was requested with the following results: TRT of 5 h 5 min, AHI of 80.1, predominantly obstructive (59.9), ODI of 82.4, and CT90 of 64.3%. With these findings, it was decided to carry out a DISE, which showed, according to the VOTE [1] scale, complete concentric velar–palatal collapse associated with complete lateral oropharyngeal collapse (see Supplementary Materials, Video S1). The patient was referred to a pneumologist, and CPAP with 9 mm/hg titration has been used successfully since then with a residual AHI of 0.9 events/h.

### 2.2. Case 2

The second case involves a 43-year-old male patient with a history of arterial hypertension (AHT) and obesity, BMI 28.1 kg/m^2^, who presented with snoring and episodes of apnea, and no tolerance to CPAP. Physical examination revealed pendulous grade 4 tonsils with septal deviation to the left and inferior turbinate hypertrophy. A polygraph revealed severe OSA (AHI 37.6, ODI 45%). Myofunctional evaluation showed reduced IOPI scores; 37 kps in the maximum tongue strength and 17 kps in maximum lip strength.

A DISE was carried out, in which, according to the VOTE scale, a partial lateral collapse was evident at level 1. At level 2, the palatine tonsils that extend to the vallecula, despite approaching the midline with each inspiration, clearly showed how they support the base of the tongue, preventing its anteroposterior collapse (see Supplementary Materials, Video S2).

Based on these findings, it was decided not to perform TA but to indicate continuous positive airway pressure therapy, encourage weight loss to reach 26.8 kg/m^2^, provide myofunctional therapy (MT) with Airway Gym^®^, and septoplasty with turbinoplasty. At post-operative follow-up after 12 months, a new polygraph showed a significant improvement (AHI 19.6, ODI 28, and CT90 24.9%) as well as in daytime sleepiness. The patient decided to keep on reducing weight and refused TA. He adapted to use CPAP with a titration of 6 mm/hg and a residual AHI of 0.3 events/h.

### 2.3. Case 3

The third case involves a 35-year-old male patient with a history of daily somnolence, arterial hypertension (AHT), and obesity at BMI 27.1 kg/m^2^ who presented with snoring and episodes of apnea. Physical examination revealed grade 4 tonsils with normal nose permeability. We asked for a polygraph for diagnosing severe OSA (AHI 45.1 and ODI 42.9). Myofunctional evaluation showed normal IOPI scores: 61 kps in maximum tongue strength and 31 kps in maximum lip strength.

A DISE was carried out, in which, according to the VOTE scale, displayed a complete anteroposterior collapse V2O1LT1E1. The palatine tonsils avoided a complete collapse of the velum. We performed a TA and barbed pharyngoplasty. After 5 years, the patient returned with similar DISE and polygraph results V2O2LT1E1 (AHI 41.2 and ODI 30.1%) without remarkable change in BMI (26.9 kg/m^2^) (see Supplementary Materials, Video S3). The patient complained of persistent dryness and oral discomfort since surgery. The patient was referred to a pneumologist and CPAP with 7 mm/hg titration has been used successfully since then, with a residual AHI of 0.5 events/h. All case presentations are summarized in Table 1.

## 3. Discussion

Several UA surgical techniques have been documented for treating OSA, although outcomes vary widely [2]. TA alone is a treatment option for OSA in adults, especially those with large tonsils [14,15,16,17,18,19,20].

The meta-analyses by Camacho et al. [16] and Reckley et al. [17] demonstrate that TA in the treatment of adults with OSA can have high surgical success rates (85.2%) and high cure rates (57.4%) according to Sher’s criteria (AHI reduction of more than 50% and a total AHI of less than 20 after surgery) [20] in selected patients (grade 3–4 tonsils, BMI < 30 kg/m^2^, and AHI < 30). They included 17 studies (*n* = 203) with follow-up times ranging from 1 to 15 months [5]. The mean AHI was reduced from 40.5 to 14.1, and the ESS score decreased from 11.6 to 6.1. This wide variability can be attributed to differences in patient characteristics, the AHI criteria used to define success, and length of follow-up.

Back in 2000, Verse et al. [21] presented a prospective study with 11 adults who underwent TA as a single treatment for OSA. With a follow-up time of 3 to 6 months, the surgical response rates were 80.0% in severe apneics and 100% in mild apneics. Smith et al. [13] retrospectively analyzed 34 patients with OSA who had TA performed and found that the mean AHI reduced from 31.6 to 8.1, with a 78% surgical response to treatment. Holmlund et al. [9] analyzed 28 adult patients with OSA and large tonsils (Friedman tonsil sizes 3 and 4). After TA, with a 6-month follow-up, the mean AHI was reduced from 40 to 7. The index was reduced in all patients, and 18 (64%) were cured.

However, Sjöblom et al. [19] conducted a retrospective study with long-term follow-up of 151 patients with OSA who underwent surgery between 2004 and 2018. The results showed that, after TA, the initial success rate was 61.9%, and 8 patients did not respond to treatment, while the late success rate was 47.6%, and 11 patients were non-responders [18]. Some studies report similar results ranging from 47.0% to 85.2%, showing that a large group of patients do not respond, with OSA persisting or worsening [16,19,21,22], especially in the long term.

A comparable situation occurs in children. Despite the benefits of adenotonsillectomy, residual OSA occurs in 21% to 75% of post-surgery pediatric cases [20]. Recognized risk factors for residual OSA in this population include young age (3 years), older age (>7 years), higher BMI, obesity, severity of OSA, and the presence of comorbidities such as asthma, neurological diseases, Down syndrome, and craniofacial anomalies. In a recently published paper, Zazal et al. [23] presented three cases of children with worsening OSA after adenotonsillectomy. In all three cases, the children had noteworthy medical histories: traumatic brain injury, Down syndrome, and Fragile X syndrome.

However, no publications have been made on residual OSA in adults after TA or on TH stabilizing the UA. The three cases that we are presenting show how TA in adults with OSA may not be the correct treatment in all cases, and sleep surgeons should be cautious to anticipate good results with this surgery.

A study by Nomura et al. [18] evaluated the efficacy of uvulopalatopharyngoplasty (UPPP) and post-operative period morphometric changes by tomography. One of the eleven assessed patients continued to have severe OSA after surgery due to a reduction in the posterior UA diameter caused by posterior displacement of the tongue and epiglottis. In another study, Yao et al. [24] also demonstrated decreased glossopharyngeal space after UPPP. They speculated that the resection of the tonsils, which supported the tongue, was responsible for the backward displacement of the soft tissue and hyoid bone. In addition, Wang et al. [25] published a study of 85 adult patients with OSA who underwent UPPP/TA, noting that when DISE showed concentric collapse, post-operative outcomes were significantly lower. These findings largely align with our theory, which questions whether all patients with OSA and large tonsils should be systematically operated on with TA.

Nowadays, DISE is the standard method for diagnosing and treating OSA, and it is the only method for understanding the levels and mechanisms of sleep obstruction [1,2,26,27]; however, the latest guidelines and consensus indicate that patients with grade 3–4 tonsils should not undergo this assessment before surgery, allowing direct surgical indication [1,11]. As a result, many patients are excluded from this assessment, which can lead to the morphological and functional consequences described above.

**Figure 4:**
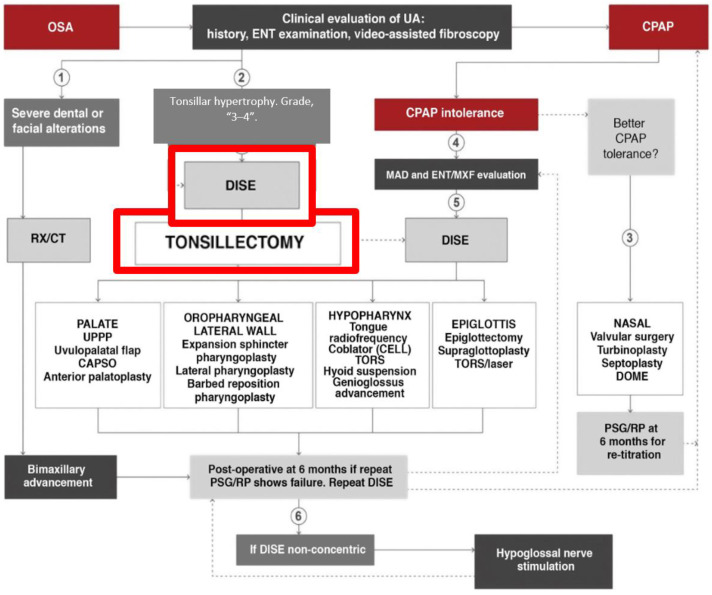
The indication of the new algorithm for surgical treatment of obstructive sleep apnea (OSA) in adults follows precise and customized medicine. TA is recommended with previous DISE.

However, some authors have shown that TA alone can be enough to treat OSA, avoiding the association of soft palate surgery. In a retrospective study on 33 patients with OSA and grade 3 or 4 tonsils treated with TA, Baudouin et al. [28] compared TA efficacy alone and associated with soft palate surgery after DISE findings. The success rate was 72.2% after TA alone and 60% when associated with soft palate surgery, a non-significant difference. The authors concluded that soft palate surgery with TA does not seem mandatory to increase the success rate after TA alone. More recently, Sundman et al. [29] compared the effectiveness of TA versus modified UPPP in patients with tonsillar hypertrophy and OSA. This randomized clinical trial evaluated surgical therapies in patients with tonsils grade 3–4. The study’s findings suggest the differential benefits of the two procedures and provide evidence that the modified UPPP was not superior to isolated TA in selected patients. However, they considered TA as the sole procedure in selected patients based on patient-specific anatomical factors and the severity of OSA. Carrasco-Llatas. [26] discuss the role of DISE as a crucial tool for surgical planning in patients with OSA. They highlighted how DISE enables personalized assessment of UA collapse patterns during sleep, facilitating more precise surgical decision-making. By visualizing the areas of obstruction during induced sleep, sleep surgeons can identify the specific anatomical sites responsible for airway blockage, leading to tailored interventions such as UPPP, TA, or other airway modifications. This study underscores the importance of DISE in optimizing surgical outcomes and minimizing unnecessary or ineffective procedures in OSA treatment. In our series, DISE has been a fundamental tool to examine reasons for failure and possible alternative rescue therapies. We disagree with international consensus [1] where DISE is not recommended in all cases with TH. We recommend a new algorithm, as seen in the figure below (Figure 4).

Qi et al. [30] published a systematic review and meta-analysis examining surgical failures identified through DISE. The study evaluates how DISE can guide surgical planning and assesses the factors contributing to unsuccessful surgical outcomes despite anatomical preoperative evaluations. They concluded that UAS guided by DISE in patients with OSA had a low failure rate of 37%. DISE can identify obstruction sites associated with surgical failure and guide single-level and multi-level surgeries.

Iannella et al. [31] suggest a comprehensive and collaborative effort based on an international experts panel, to provide evidence-based guidance on what constitutes surgical failure, the factors contributing to it, and strategies to mitigate it. They suggested that no surgical treatment is 100% effective, and many have shown limited clinical application due to lower effectiveness in reducing hypopnea/apnea events or a higher incidence of related comorbidities. UAS should be carefully selected based on the patient’s anatomy, clinical characteristics, sites of obstruction, type of collapse, clinical severity of OSA, and the potential complications of each surgical technique.

Kumar et al. [32] suggested a new way to select patients based on upper airway (UA) collapsibility metrics, which may explain why many patients with OSA have persistent symptoms after surgery. They analyzed 20 patients with OSA, adding to the DISE the luminal pressure measurements obtained with a catheter in the velopharynx. Three phenotypes were obtained: inspiratory collapse (phenotype 1), expiratory collapse (phenotype 2 = palatal prolapse), and a primarily stable airway during inspiration and expiration that collapsed as CPAP was reduced (phenotype 3). They conclude that velopharyngeal compliance is not significantly different at peak inspiration and peak expiration, including a new variable to study before considering treatment, and additional studies are needed to understand the biomechanics of palatal prolapse and its importance in OSA pathophysiology and therapy selection.

The occurrence of the three reported cases in men does not appear to be a coincidence, as the prevalence of OSA is significantly higher in men than in women. Various cohort studies have shown that the prevalence of OSA in men varies considerably, ranging from 4% to 59% for mild OSA (AHI ≥ 5) and from 6.5% to 30% for moderate to severe OSA (AHI ≥ 15). Therefore, the gender distribution observed in the cases presented in the manuscript reflects this general trend in the population [33,34].

TA alone is a treatment option for OSA in adults, especially those with large tonsils. However, before deciding on the therapeutic approach, it is essential to assess other anatomical factors that also significantly influence the severity of snoring and OSA. These factors include increased BMI, large neck circumference, facial skeletal abnormalities, tongue size, hypertrophy of the lingual tonsil, and nasal obstructions (such as polyps or rhinitis), among others. All these elements must be carefully considered to ensure an appropriate and effective treatment. In the patients presented in this document, all these variables were thoroughly evaluated to determine the most suitable treatment [35].

We understand the limited number of cases presented and the absence of a prospective randomized clinical trial to assure our reflections. Also, we emphasize the absence of preoperative DISE in the first case and the lack of post-operative DISE in the second one to explain the pathophysiology of this palatal collapse. In the first case, we did not perform DISE following the international consensus algorithm, and in the second case, we used the information obtained in the first case and shared it with the patient who decided not to go ahead with TA. In the third case, we had the opportunity to repeat the DISE after five years of surgery to analyze the reason for our failure. We are aware of the limitations of the actual knowledge about velopharyngeal physiology and the need for further research to provide it.

## 4. Conclusions

Limited data exist on isolated TA as a treatment for OSA in adults, and high-level evidence studies are needed to reach definitive conclusions. Further research is essential to better identify the characteristics of patients who do not respond to TA and, based on this, establish clear criteria for selecting candidates who would benefit significantly from surgery. In our opinion, performing a DISE before surgery should be standard practice to optimize outcomes and minimize the risk of treatment failure.

## Figures and Tables

**Table 1:** Presentation of cases.

Case	Age	Gender	Severity of OSA	Treatment	Results/Impacts on OSA
1	24	Male	Severe OSA (AHI 80)	Tonsillectomy	Worsening in the AHI (80.2) and symptoms.
2	43	Male	Severe OSA (AHI 37.6)	Septoplasty CPAP Weight loss MT	Improvement in the AHI (19.6) and symptoms.
3	35	Male	Severe OSA (AHI 45.1)	Barbed pharyngoplasty Tonsillectomy	No significant changes in the AHI (41.2) and new symptoms have emerged.

AHI: Apnea–Hypopnea Index; OSA: obstructive sleep apnea, MT: myofunctional therapy, CPAP: continuous positive airway pressure. See supplementary materials.

## Data Availability

The data presented in this study are available on request from the corresponding author.

## Supplementary Materials

The following supporting information can be downloaded at: https://www.mdpi.com/article/10.3390/jcm13216456/s1, Video S1: DISE after TA; Video S2: Stent phenomenon; Video S3: Pre- and 5 years post-operative DISE after TA and barbed pharyngoplasty.

## List of Abbreviations

AHI	Apnea–Hypopnea Index
AHT	Arterial hypertension
BMI	Body mass index
CPAP	Continuous positive airway pressure
CT90	Percentage of the time spent below 90% O_2_ saturation
DISE	Drug-induced sleep endoscopy
ESS	Epworth sleepiness scale
MT	Myofunctional therapy
ODI	Oxygen Desaturation Index
OSA	Obstructive sleep apnea
SDB	Sleep-disordered breathing
TA	Tonsillectomy
TH	Tonsillar hypertrophy
TRT	Total recording time
UA	Upper airway
UAS	Upper airway surgery
UPPP	Uvulopalatopharyngoplasty
VOTE	Velum Oropharynx Tongue Epyglottis
